# “Beyond the Sad Eyes”: A Pilot Study on Behavioural and Physiological Indicators in Shelter Dogs Exhibiting Depression-like Behaviour

**DOI:** 10.3390/ani16132079

**Published:** 2026-07-05

**Authors:** Sara Boero, Clara Palestrini, Greta V. Berteselli, Alice Garegnani, Tanja Peric, Isabella Pividori, Alberto Prandi, Michela Minero, Silvia M. Mazzola, Simona Cannas

**Affiliations:** 1 Department of Veterinary Medicine and Animal Science, University of Milan, Via dell’Università 6, 26900 Lodi, LO, Italygreta.berteselli@gmail.com (G.V.B.);; 2 Department of Agricultural, Food, Environmental and Animal Sciences, University of Udine, Via Sondrio 2B, 33100 Udine, UD, Italy; tanja.peric@uniud.it (T.P.);

**Keywords:** animal welfare, depression-like patterns, inactivity, accelerometry, allopregnanolone, stress biomarkers, hair

## Abstract

Shelter dogs may experience long-term environmental and social stressors that can affect their behaviour and welfare. Some individuals show reduced activity, low responsiveness to environmental stimuli, and limited interaction with their surroundings. This pilot study investigated behavioural patterns and allopregnanolone concentrations in shelter dogs exhibiting these behavioural characteristics. Ten shelter dogs were enrolled and divided into two groups: five dogs showing depression-like patterns and five matched control dogs. Each dog wore a three-axis accelerometer for 30 days to quantify activity levels. Behavioural observations were conducted using video recordings, and hair samples were collected at baseline and after 30 days to assess allopregnanolone concentrations as a potential stress marker. Dogs in the case group showed significantly longer resting time than controls (*p* ≤ 0.05), indicating reduced activity levels. Trends toward lower levels of exploratory and social behaviours were also observed, although the differences were not statistically significant. Allopregnanolone concentrations ranged from 0.6 to 4.6 pg/mg and showed considerable inter-individual variability, with no significant differences detected between groups. These findings provide preliminary evidence of behavioural and physiological alterations in shelter dogs displaying depression-like patterns. However, further studies with larger populations are needed to validate these findings and improve welfare assessment in shelter environments.

## Simple Summary

Dogs living in animal shelters may experience long-term stress that can affect their behaviour and welfare. This pilot study investigated whether dogs exhibiting depression-like behaviours, such as low activity and reduced responsiveness to their environment, differed from other shelter dogs in activity levels and in a physiological marker of stress regulation. Ten shelter dogs were monitored for one month using activity sensors, behavioural observations, and hair sample analysis. Five dogs displaying depression-like patterns were compared with five control dogs living under similar shelter conditions. Dogs in the depression-like group spent more time resting than control dogs. Hair concentrations of allopregnanolone, a neuroactive steroid involved in stress regulation, tended to be higher in these dogs, but no significant differences were found between groups. These preliminary findings suggest that combining behavioural observations, activity monitoring, and physiological measures may provide a more comprehensive assessment of welfare in shelter dogs and help identify individuals experiencing chronic stress or depression-like states.

## 1. Introduction

In animals, depression-like states are generally described as clusters of behavioural and physiological alterations, including reduced activity, apathy, social withdrawal, and changes in sleep and feeding patterns, and psychomotor alterations, as well as other conditions such as anxiety and social isolation [1]. These conditions have been reported in several species, including rodents, horses, primates, and dogs.

Behaviours related to despair, learned helplessness, apathy, anxiety, anhedonia, social interaction, and cognitive deficits are evaluated [1].

Learned helplessness, defined as a perceived lack of control over adverse events, has been widely studied in animal models and is associated with stress-related behavioural and physiological changes [2]. These include dysregulation of the hypothalamic–pituitary–adrenal (HPA) axis and alterations in glucocorticoid concentrations [3,4,5,6].

Inactivity, described in mice as an “awake but motionless animal,” is considered a depressive-like symptom. These animals remain alert yet motionless in their cages during the day [3]. Apathy, on the other hand, is considered a deficit in the willingness to engage in goal-directed behaviour [7]. A study on anhedonia was conducted in kennelled dogs, in which it was hypothesised that their ability to experience pleasure might be reduced due to chronic stress from prolonged social, dietary, and spatial restrictions [8].

Shelter environments expose dogs to multiple potential stressors, including social isolation, confinement, unpredictable stimuli, and reduced environmental enrichment. These conditions may lead to behavioural changes such as reduced activity, stereotypies, altered exploratory behaviour, and modifications to the sleep–wake cycles [9,10,11,12].

From a physiological perspective, allopregnanolone (3α-hydroxy-5α-pregnan-20-one) is a neuroactive steroid involved in stress modulation and emotional regulation. Variations in its concentration have been associated with stress-related conditions (anxiety, panic, and depression) in human and animal models, although its role in dogs remains poorly studied. The GABA receptor complex has been documented as the primary target of allopregnanolone, with most of its inhibitory action mediated through GABA enhancement or direct activation of GABA currents. Some studies have also described the involvement of multiple other targets, including brain-derived neurotrophic factor (BDNF), glutamate, dopamine, opioids, oxytocin, and calcium channels [13].

Hair cortisol concentration is widely recognised as a non-invasive marker of cumulative hypothalamic–pituitary–adrenal (HPA) axis activity and long-term stress exposure in dogs. However, cortisol primarily reflects glucocorticoid output and may not fully capture other neuroendocrine pathways that are potentially involved in stress regulation, behavioural inhibition, and affective modulation. In this context, allopregnanolone may be an exploratory, complementary biomarker rather than an alternative to cortisol.

Studies in mice have explored the correlation between allopregnanolone and the HPA axis, revealing that allopregnanolone synthesis contributes to stress resilience [14]. Through its modulation of GABAergic signalling, allopregnanolone may contribute to the regulation of HPA axis feedback and to the recovery of homeostasis after stress exposure. Therefore, its assessment may provide complementary information on neurosteroid-mediated stress regulation, particularly in subjects exposed to chronic environmental and social stressors. Nevertheless, the biological interpretation of allopregnanolone concentrations in dogs, especially in hair, remains poorly established, and its relationship with depression-like behavioural patterns in this species should be considered exploratory. So far, allopregnanolone has been investigated mainly in plasma samples from humans [15], horses [16], and rodents [17], particularly in the context of depressive disorders, neurodegenerative diseases, and stress reactivity. Recently, hair has also been proposed as an interesting sample for studying allopregnanolone [18]. Hair is a non-conventional matrix that has significant advantages, including the following: sampling is simple, can be performed by a single operator, and is not invasive or painful for the individual that is being sampled; sample storage can be in a paper envelope, which must be kept away from direct light and at room temperature; and hair may provide a more stable and cumulative measure of steroid exposure over time, being less affected by short-term hormonal fluctuations than blood or saliva samples. Furthermore, hair is a retrospective and cumulative matrix, meaning it can provide historical information about hormone concentrations with a lag time of ~2 weeks [19]. Ellero et al. (2025) [20] used hair sampling to assess allopregnanolone concentrations in newborn foals. They found that reduced foal allopregnanolone concentrations and a lower foal-to-mare allopregnanolone ratio in sick animals could serve as potential biomarkers of prenatal disease during late pregnancy.

In the present study, hair allopregnanolone was therefore selected as an innovative, exploratory neurosteroid marker to complement behavioural observations and accelerometer-based activity monitoring in shelter dogs. This choice was based on its potential involvement in stress modulation and affective regulation, while acknowledging that reference values and physiological variability for hair allopregnanolone in dogs are not yet available. Accordingly, the assessment of hair allopregnanolone in this pilot study should be interpreted as hypothesis-generating and not as a validated biomarker of canine depression-like states.

This study aimed to investigate behavioural and physiological characteristics of shelter dogs exhibiting putative depression-like patterns, using a combination of accelerometers, behavioural observations, and hair allopregnanolone analysis. Additionally, the study aimed to compare these parameters between dogs with and without these patterns within the same shared environment. We hypothesized that dogs exhibiting putative depression-like patterns would show reduced activity levels as well as behavioural and physiological differences compared with control dogs.

## 2. Materials and Methods

### 2.1. Data Collection and Animal Sampling

A case–control study was conducted between March 2022 and December 2023 in two dog shelters located in northern Italy. Dogs were selected by veterinarians with expertise in animal behaviour based on behavioural assessments. The veterinarians also performed clinical evaluations to exclude medical conditions that could affect behaviour; in senior dogs, DISHA syndrome (canine cognitive dysfunction syndrome) was specifically ruled out using the validated Canine Dementia Scale (CADES). A total of 10 dogs (5 males and 5 females), aged between 6 and 15 years, were included in the study. Five dogs displaying depression-like behavioural characteristics were assigned to the case group, while five dogs without these characteristics served as controls. Dogs allocated to the depressive-like group had been previously diagnosed by a veterinarian specialized in Applied Ethology and Animal Welfare and a board-certified veterinary behaviourist (Diplomate of the European College of Animal Welfare and Behavioural Medicine, Behavioural Medicine subspecialty), based on a comprehensive clinical behavioural assessment. The assessment consisted of a standardized clinical behavioural examination lasting approximately 90 min and included direct observation of the dog’s spontaneous behaviour, responsiveness to environmental and social stimuli, exploratory activity, posture, facial expression, and interactions with humans and conspecifics.

Dogs were included in the depressive-like group when they consistently exhibited persistent inactivity, markedly reduced responsiveness to external stimuli, limited spontaneous exploration, reduced social interactions, and prolonged maintenance of the same resting posture throughout the 90 min behavioural examination. Before behavioural classification, medical conditions that could account for reduced activity were excluded through clinical evaluation. Control dogs underwent the same behavioural assessment but did not exhibit these behavioural characteristics, maintaining normal levels of responsiveness, spontaneous activity, exploratory behaviour, and social interaction. The two groups were matched for sex, age, body weight and length of stay in the shelter.

Both shelters provided comparable housing and management conditions. Dogs were housed in individual pens with indoor (night) and outdoor (day) areas, both of which had similar structural features, including solid walls, adequate ventilation, and access to natural light. Pens were equipped with standardised resources, such as water supply, food bowls, resting areas, and heating systems. Dogs included in the study were housed in the same kennel row at each shelter under comparable environmental conditions. They were located away from the main entrance and in designated off-leash exercise areas. This reduced potential variability in exposure to external stimuli such as visitor activity, wind, and direct environmental changes.

Dogs in both facilities had regular access to outdoor exercise areas. They were routinely exposed to similar daily management practices, including feeding schedules, cleaning procedures, and interactions with staff and volunteers. Opportunities for physical activity and environmental enrichment (e.g., free-run areas and walks) were consistently provided across both shelters.

These similarities allowed for the minimisation of environmental variability between the two study sites.

In addition, potential confounding variables, including age, sex, and duration of stay in the shelter, were taken into account during group allocation to maximise comparability between case and control groups (Table 1).

The dogs were selected from the two facilities based on reports from the shelter managers and evaluations by the veterinary behaviourists working in the shelters (6 dogs from one shelter and 4 from the second). The inclusion criteria are listed in Table 2.

**Figure 1:**
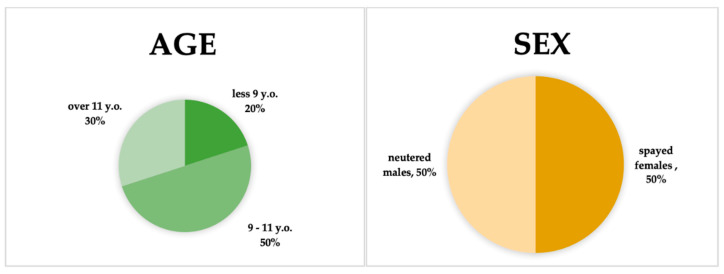
Age ranges and sex of dogs included in the study.

The age ranges of the animals and their reproductive status are shown in the graphs in Figure 1.

### 2.2. Activity Monitoring

Each dog was fitted with a PitPat activity monitor (three-axis accelerometer) for 30 consecutive days. A secure Velcro attachment was used to attach the 32 × 32 × 15 mm device to a collar. Dogs unaccustomed to wearing a collar underwent a one-week habituation period before the sensor was applied. The device recorded time spent in different activity states, including resting, walking, running, and playing.

### 2.3. Behavioural Analysis

Video recordings were conducted for one hour per day over five consecutive days at both baseline (T0) and after 30 days (T1). The 2 GoPro cameras were positioned to capture both resting and active areas of the pen, both set to wide-angle view. Recordings began after the first meal of the day to maintain consistency across different shelters. During the recording period, staff and volunteers did not enter the pen.

Data collected from the PitPat-Dog activity monitor were downloaded via a mobile application, enabling analysis of movement duration and type (walking, running, or playtime) over the 30-day study period, as well as time spent resting (distinguishing between passive time and actual rest).

Behavioural data were analysed using BORIS software (v. 8.22.4). An ethogram was developed based on previous literature and adapted to the shelter context. The behaviours analysed are categorised in Table 3.

### 2.4. Physiological Analysis—Allopregnanolone in Hair

Hair samples were collected at T0 and after 30 days (T1) using a shave-reshave technique from an area of approximately 3 × 3 cm, either from a front or hind limb [21]. The hair samples were stored in paper envelopes and kept in the dark at room temperature until processed.

Before the quantification of allopregnanolone by the Enzyme Immunoassay method using a commercial kit DetectX^®^ from Arbour Assays Inc (Product number: K061-H5, Ann Arbour, MI, USA), the sample was washed and extracted as described by Peric et al. (2023) [18], a study in which the kit was also validated for hair analysis. In detail, 100 mg of hair was rinsed with 5 mL of ultrapure water, then washed with 5 mL of isopropanol to remove surface steroids. Steroids were extracted from the hair using 3 mL of methanol in vials incubated at 37 °C for 16 h. The methanolic extract in the vial was evaporated under an air-stream suction hood. The dry residue from the hair extracts was dissolved in 0.35 mL of the allopregnanolone enzyme immunoassay kit assay buffer (previously diluted following the manufacturer’s instructions). The allopregnanolone assay was performed according to the manufacturer’s instructions. Absorbance was read at 450 nm using a microplate reader (EnSight Multimode Plate Reader, Perkin-Elmer Life Science, Boston, MA, USA). The assay sensitivity was 50.4 pg/mL. The intra- and inter-assay coefficients of variation were 8.0% and 11.2%, respectively.

### 2.5. Statistical Analysis

Statistical analyses were performed using IBM SPSS Statistics version 28 (IBM Corp., Armonk, NY, USA). Preliminary exploratory analyses were conducted through visual inspection of histograms and boxplots to assess the data distribution and identify potential outliers.

Given the limited sample size and the non-normality observed in several variables, non-parametric statistics were considered the most appropriate analytical approach. Therefore, comparisons between the case and control groups were performed using two-tailed Mann–Whitney U tests.

Descriptive statistics are reported as mean ± standard deviation (SD), together with minimum and maximum values and 95% confidence intervals where appropriate. Effect sizes for Mann–Whitney comparisons were calculated using r values derived from the standardized test statistic (r = Z/√N). Statistical significance was set at *p* ≤ 0.05.

Behavioural coding was performed by trained observers using BORIS software (v. 8.22.4). To assess inter-rater reliability, 20% of the video recordings were independently coded by two observers. Inter-observer agreement exceeded 85%, indicating satisfactory reliability of behavioural scoring.

## 3. Results

### 3.1. Analysis of Pitpat-Dog Activity Monitor Motion Sensors

The results of the motion sensor analysis for cases and controls in the shelter are presented in Table 4. “Resting” was defined as the dog lying down with its head fully supported on a surface, whereas “passive time” was defined as a stationary state (either sitting or lying down) in which the dog remained inactive but without the head resting on a surface. The activity type and duration algorithm identifies walking and running based on the dog’s characteristic gait patterns, with classification determined by movement intensity. Activity was evaluated over 5 min epochs, and the predominant behaviour within each epoch was assigned as the representative activity for that epoch. Frequent alternation between walking, running, and stopping within the same epoch is classified as play behaviour, as it reflects typical locomotor patterns associated with play in dogs.

**Figure 2:**
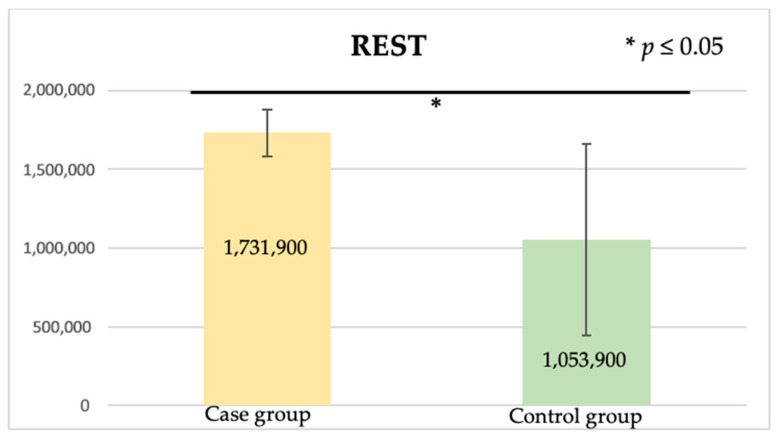
Results, in seconds, of the time spent resting as analysed with the PitPat-Dog activity monitor movement sensor in the case and control groups.

As shown in Figure 2, dogs in the case group had significantly longer resting times than those in the control group, indicating reduced activity levels (*p* ≤ 0.05).

Non-significant trends were observed in other activity parameters, with lower levels of walking, running, and playing in the case group. It was found that the dogs in the case group spent less time walking (1277 ± 1005.9), playing (235 ± 222), and running (49 ± 69.05) compared to the controls (Table 4).

### 3.2. Analysis of Behavioural Parameters of the Case and Control Group

Behavioural analysis revealed non-significant differences between groups. However, dogs in the case group showed reduced exploratory behaviours (e.g., sniffing) and lower responsiveness to environmental stimuli. In contrast, control dogs exhibited higher levels of interaction with the environment and greater behavioural variability. The behavioural analysis is summarised in Table 5.

### 3.3. Allopregnanolone in Hair Samples

Hair sampling was performed at T0 and 30 days later (T1) for all subjects in both groups (Table 6).

Due to the damage to one sample, it was not possible to perform the T1 analysis for one dog in the control group.

**Figure 3:**
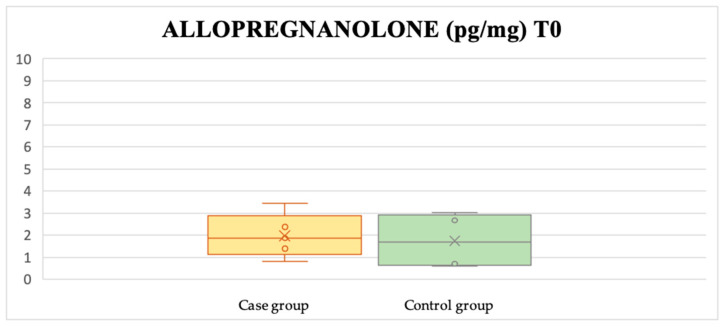

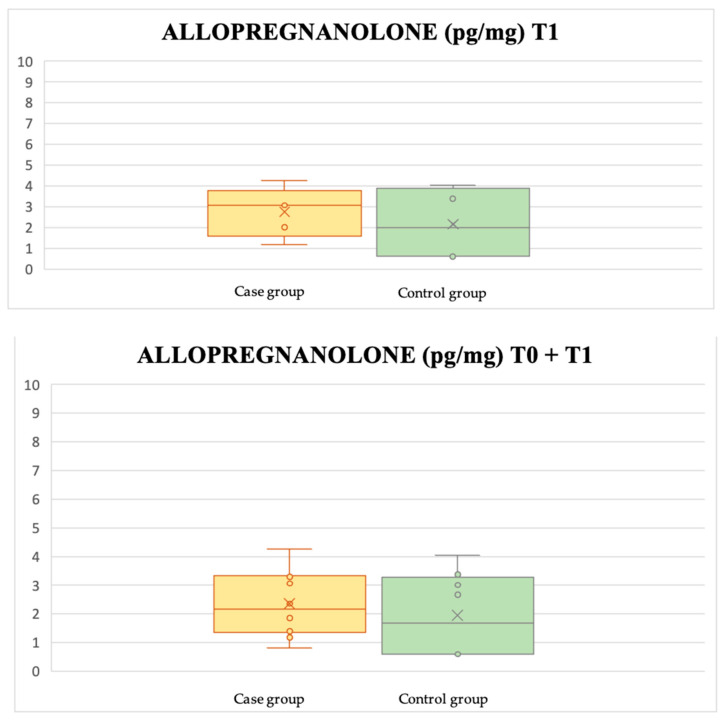
Box plot of allopregnanolone concentrations at T0 and T1 in the two groups, obtained through hair sample analysis.

Allopregnanolone concentrations ranged between 0.6 and 4.6 pg/mg and showed considerable inter-individual variability; however, there was no statistically significant difference between groups (Figure 3, Appendix A).

## 4. Discussion

The present pilot study investigated behavioural and physiological characteristics of shelter dogs exhibiting behaviours that may be associated with a depression-like pattern, integrating activity monitoring, behavioural observations, and neurosteroid analysis. While the limited sample size requires cautious interpretation, the findings provide preliminary insights into behavioural alterations associated with long-term sheltering conditions. Importantly, the physiological component of the study should be interpreted as exploratory, as allopregnanolone was investigated as a candidate neurosteroid marker rather than as a validated biomarker of canine depression-like states or chronic stress.

Dogs included in the case group had generally spent a substantial portion of their lives in the shelter environment. As dogs in both groups were generally older, age-related factors, including reduced mobility, sensory decline, and possible musculoskeletal conditions such as osteoarthritis, may have contributed to the observed behavioural patterns. Prolonged exposure to confined conditions, reduced environmental stimulation, and limited social interactions have previously been associated with behavioural and physiological changes indicative of chronic stress in dogs [10,11,22]. In this context, the behavioural patterns observed in the case group may reflect a combination of age-related changes and adaptive or maladaptive responses to long-term environmental constraints.

Therefore, the observed phenotype should not be interpreted as a direct equivalent of clinical depression, but rather as a depression-like or apathetic-like behavioural profile characterised by reduced activity, lower environmental engagement, and decreased responsiveness.

A key finding of this study was a significantly increased resting time in the case group compared with the control group. Reduced activity levels, particularly prolonged inactivity while awake, have been described in several species as a potential indicator of altered affective states. This behavioural pattern may be associated with apathy or reduced motivation to interact with the environment, which are commonly discussed in relation to depression-like states [1]. In particular, these dogs exhibit a form of inactivity, spending most of their time awake but motionless in their environment [23], and behaviours such as learned helplessness, where the individual realises they have no control over unpleasant and harmful events, rendering their actions useless and leaving them powerless [3,5,6]. As a result, the animal experiences stress over which it has no control [2].

However, it should be emphasised that inactivity alone cannot be considered a definitive indicator of such a condition, as it may also result from age, health status, or environmental factors.

In fact, sedentary behaviour, in which the animal remains lying down, unresponsive to stimuli, and passive, can be considered a behavioural change associated with stress caused by confinement and limited environmental and social stimulation in the shelter [11,24]. In particular, the longer rest periods observed in the case group and the overall reduction in activity may be consistent with apathy and decreased responsiveness potentially associated with chronic stress; however, alternative explanations, including age-related factors, physical discomfort, or other non-pathological conditions, cannot be excluded. Activities such as running, walking, and play behaviour were observed more frequently in the control group; however, these differences were not statistically significant and should therefore be interpreted as trends rather than confirmatory evidence of higher activity levels.

Although not statistically significant, several behavioural trends emerged from video analysis. Dogs in the case group showed reduced exploratory behaviours, such as sniffing, and lower responsiveness to environmental stimuli. Olfactory activity directed towards the environment, the air, other dogs, and people is part of a dog’s normal exploratory behaviour [23]. Olfactory signals provide information about the surroundings [25,26]. Additionally, olfactory activity has a calming effect on the animal [27]. Dogs that engage in olfactory activities exhibit a more optimistic attitude than those that do not [28]. The reduction or absence of this behaviour observed in the case group may therefore be associated with behavioural inhibition and reduced exploratory activity, potentially related to apathy and stress [11]. Nevertheless, because these differences did not reach statistical significance, they should be considered descriptive behavioural trends that require confirmation in larger samples.

These findings are consistent with previous studies suggesting that decreased interaction with the environment may reflect reduced engagement or motivational states. Similarly, lower frequencies of social and activity-related behaviours, including movement between areas and tail wagging, were observed in the case group, supporting the hypothesis of reduced behavioural activation.

However, in shelter environments, reductions in activity and exploratory behaviour have been described as behavioural changes potentially associated with stress [11]. The lower frequency of position changes observed in the case group may reflect decreased activity, reduced attention to the external environment, and limited exploratory behaviour, potentially related to stress. Nevertheless, alternative explanations should also be considered, including age-related factors, habituation to kennel housing, and the possibility that some dogs may be well adapted to the housing conditions without necessarily experiencing elevated stress levels.

Tail wagging was classified into normal wagging, in which the dog moves its tail with varying intensity, and high-tail wagging, in which the tail is held high with short, rigid movements [23]. An increase in tail wagging may indicate heightened excitement in response to external stimuli [29]. Video analysis showed that normal tail wagging occurred in both groups, but it was more frequent in the control group, particularly when a handler passed by the pen. High-tail wagging was observed in only a few cases in the control group, usually directed at a passing dog. This behaviour may thus be interpreted as a sign of sociability towards handlers and, in the latter case, as an expression of attention towards another dog [23]. The reduction in this behaviour in the case group may indicate reduced behavioural activation and decreased reactivity to external stimuli [11].

Stereotyped behaviours associated with prolonged stays in pens are considered signs of chronic stress and poor welfare [9,30]. The absence of such behaviours in the case group should be interpreted with caution, as recordings were conducted for only 1 h per day. Thus, the presence of these behaviours at other times of the day cannot be ruled out.

The analysis of allopregnanolone concentrations showed differences in mean concentrations between the case and control groups; however, these differences were not statistically significant.

Allopregnanolone is a neuroactive steroid involved in stress modulation and emotional regulation. Alterations in allopregnanolone concentrations have been reported in humans exposed to stress and in stress-related conditions such as anxiety, panic, and depression [13]. During acute stress, allopregnanolone concentrations may increase, contributing to the regulation of the hypothalamic–pituitary–adrenal (HPA) axis and the restoration of homeostasis [13,14,31,32,33].

Conversely, chronic or repeated stress exposure may be associated with altered neurosteroid regulation, potentially impairing the ability to modulate stress responses. This mechanism is biologically plausible because allopregnanolone acts mainly through GABAergic signalling, which is involved in inhibitory control, stress recovery, and affective regulation.

The rationale for measuring allopregnanolone in the present study was therefore not to replace cortisol, but to explore a complementary neuroendocrine pathway that may be involved in stress-related behavioural inhibition and affective dysregulation. Hair cortisol concentration remains the more established biomarker for assessing cumulative HPA axis activity and long-term stress exposure in dogs. Cortisol primarily reflects glucocorticoid output, whereas allopregnanolone may reflect neurosteroid-mediated modulation of stress responses, particularly through GABA-A receptor activity. In this sense, the two biomarkers should be considered complementary rather than interchangeable.

The relationships among allopregnanolone, cortisol, and serotonin pathways under chronic shelter stress remain largely hypothetical in dogs. Cortisol reflects HPA axis activation, while allopregnanolone may influence HPA axis feedback through GABAergic mechanisms. Serotonergic pathways are also involved in mood, motivation, behavioural inhibition, and stress coping; however, serotonin or serotonin-related metabolites were not measured in this study. Therefore, no direct conclusions can be drawn regarding serotonergic involvement. Future studies combining hair cortisol, allopregnanolone, and behavioural or neurochemical indicators related to serotonin would be useful to better clarify the physiological framework underlying depression-like patterns in shelter dogs.

In the present study, no statistically significant differences in allopregnanolone concentrations were observed between groups. Given the variability of the data and the limited sample size, studies including a larger number of subjects may help to better evaluate potential group differences and further clarify the role of allopregnanolone in canine stress and affective states. Furthermore, reference values for hair allopregnanolone concentrations in dogs are currently unavailable, and physiological variability related to age, sex, reproductive status, health status, coat characteristics, and chronic stress exposure remains poorly characterised. Accordingly, the concentrations observed in the present study should be interpreted only as preliminary values for this specific sample, not as reference intervals.

### Study Limitations

Several methodological limitations should be acknowledged. First, the small sample size substantially limits statistical power and increases the risk of Type II error, potentially masking meaningful differences between groups. This limitation is particularly relevant for the interpretation of allopregnanolone, given the novelty of this biomarker in dogs and the considerable inter-individual variability observed in the present study. Second, behavioural observations were based on a limited recording period (one hour per day over five days), which may not fully capture the range and variability of behaviours expressed throughout the day. Third, activity data were collected using collar-mounted accelerometers, which may be less sensitive to certain postural changes and low-intensity movements, potentially affecting the precision of activity measurements.

Furthermore, the classification of dogs into case and control groups was based on behavioural assessment rather than validated diagnostic criteria, introducing a degree of subjectivity. Although all dogs were assessed using a standardized clinical behavioural examination performed according to predefined behavioural criteria, validated quantitative cut-off values for depressive-like behaviour are currently unavailable. Therefore, classification necessarily relied on expert clinical judgment, which may limit the exact reproducibility of the inclusion criteria across independent studies. An additional limitation is the absence of a specific behavioural paradigm designed to assess motivational state or anhedonia. Although increased inactivity and resting behaviour may be consistent with a depression-like or apathetic-like condition, these measures alone cannot distinguish reduced motivation from adaptation to a low-stimulation environment. Future studies would benefit from incorporating validated assessments of affective state and motivation, such as cognitive bias paradigms or reward-based preference tests.

Potential confounding factors such as age, individual history, and duration of shelter stay may also have influenced the results. In particular, the dogs classified as cases were older than controls, and age-related factors such as reduced mobility, joint discomfort, or sensory decline may have contributed to lower activity levels and increased resting behaviour. Therefore, the observed inactivity should not be interpreted exclusively as evidence of a depression-like condition. Although clinical evaluations were performed to exclude overt medical conditions, subclinical pain or mild musculoskeletal impairment cannot be completely ruled out and may have influenced activity patterns.

Finally, the study did not include a traditional marker of hypothalamic–pituitary–adrenal (HPA) axis activity, such as hair cortisol. The inclusion of such measures would have provided complementary information on stress physiology and a useful reference for interpreting the allopregnanolone findings. The use of a larger and more diverse sample, combined with longitudinal designs and validated behavioural assessment tools, would strengthen the reliability of future findings.

Despite these limitations, this study highlights the potential value of combining behavioural and physiological measures to investigate affective states in shelter dogs. The integration of accelerometer-based monitoring and non-invasive biomarkers may represent a promising approach for improving welfare assessment in kennel environments.

## 5. Conclusions

This pilot study provides preliminary and exploratory data on potential behavioural and physiological differences between shelter dogs exhibiting putative depression-like patterns and control dogs. Increased resting time was the most consistent observation; however, this finding should be interpreted cautiously and cannot be considered a specific or validated indicator of altered affective states.

Given the limited sample size and methodological constraints, these results should not be interpreted as evidence supporting the use of the present methodology for welfare assessment or diagnosis. Rather, they highlight possible associations that require further investigation.

Future studies with larger sample sizes, longitudinal designs, and validated behavioural paradigms, including objective measures of motivation and reward sensitivity, are necessary to confirm these preliminary observations. In this context, further validation is needed before non-invasive tracking approaches can be applied specifically to the assessment of canine depression-like states.

## Figures and Tables

**Table 1:** List of dogs included in the study.

Name	Group	Shelter	Breed	Coat	Birth	Sex	Weight
**Ivry**	Case	A.DI.CA	mix breed	brown	01/01/14	FS	28 kg
**Glitter**	Case	A.DI.CA	mix breed	black	01/01/14	MN	30 kg
**Cento**	Case	A.DI.CA	maremma sheepdog	white	01/01/13	MN	34 kg
**Ian**	Case	Parco Canile	catalan sheepdog	cream	14/08/10	MN	28 kg
**Irina**	Case	Parco Canile	sheepdog mix	fawn	01/01/08	FS	28 kg
**Parma**	Control	A.DI.CA	mix breed	cream	01/01/12	FS	26 kg
**Rocky**	Control	A.DI.CA	mix breed	black and tan	01/01/14	MN	26 kg
**Irlanda**	Control	A.DI.CA	mix breed	fawn	01/01/10	FS	20 kg
**Asia**	Control	Parco Canile	amstaff	white and chestnut	15/05/16	FS	29.2 kg
**Maicol**	Control	Parco Canile	mix breed	white and brown	15/10/17	MN	21.4 kg

FS = female spayed. MN = male neutered.

**Table 2:** Group division and inclusion criteria.

Cases	Controls
Inactivity or low activity in kennel	Activity in kennel
Lack of reactivity to stimuli	Reactivity to stimuli
Indifference to the environment	Clinically healthy, without specific debilitating organic pathologies
Clinically healthy, without specific debilitating organic pathologies	

**Table 3:** List of behaviours subject to analysis and their respective categories.

Behaviour	Type	Category
Sniff the air	point event	sniffing
Sniff the food	point event	sniffing
Sniff the environment	point event	sniffing
Indifferent to barking	state event	attitudes
Look at a stranger/volunteer	state event	attitudes
Look outside attentively	state event	attitudes
Attention	state event	attitudes
Front paws on the wall	state event	attitudes
Scratch the door	state event	activities
Drink	point event	activities
Move between sleeping/active areas	state event	activities
Trot	state event	activities
Gallop	state event	activities
Yawn	point event	activities
Turn around	point event	activities
Scratch	point event	activities
Urinate	point event	elimination
Defecate	point event	elimination
Scratch the floor	point event	elimination
Grooming	state event	grooming
Crouch	point event	positions
Lie down	point event	positions
Sit	point event	positions
Stand up	point event	positions
Wag the tail	point event	tail positions
Lick self/lick an object persistently	state event	stereotypies
Tail chasing	point event	stereotypies
Bark	state event	vocalisations
Howl	state event	vocalisations

**Table 4:** Duration (in minutes) of activities in the case and control groups as recorded by the PitPat-Dog activity monitor.

	Case Group	Control Group
walking	1277 ± 1005.9	1592 ± 1781.78
running	49 ± 69.05	103 ± 94.38
playing	235 ± 222	538 ± 475.45
passive time	12,685.2 ± 2977.35	14,962 ± 8416.63
resting	28,865 ± 2445.05	17,565 ± 10,151.78

**Table 5:** Behavioural differences between case and control dogs.

**Environmental sniffing**	Lower in the case group
**Air sniffing**	Lower in the case group
**Food sniffing**	No difference between groups
**Indifference to barking**	Higher in the case group
**Looking outside enclosure (attention)**	Lower in the case group
**Front paws on wall (attention posture)**	Lower in the case group
**Movement between kennel areas**	Lower in the case group
**Trotting/galloping**	Lower in the case group
**Drinking**	Lower in the case group
**Yawning**	Lower in the case group
**Turning**	Lower in the case group
**Scratching**	Lower in the case group
**Urination**	Lower in the case group
**Postural transitions**	Lower in the case group
**Crouching**	Higher in the case group
**Tail wagging**	Lower in the case group
**Howling**	Lower in the case group
**Jumping, licking, barking, defecation, ground scratching, door scratching, tail chasing, grooming**	No difference between groups

**Table 6:** Allopregnanolone concentrations detected through hair sample analysis at T0 and T1 in the two groups.

Name	Group	Sampling Type	Allopregnanolone (pg/mg)
Ivry	Case	T0	1.4
		T1	2.0
Glitter	Case	T0	2.4
		T1	3.3
Cento	Case	T0	0.8
		T1	1.2
Ian	Case	T0	1.9
		T1	4.2
Irina	Case	T0	3.4
		T1	3.1
Parma	Control	T0	2.7
		T1	3.4
Rocky	Control	T0	3.0
		T1	4.0
Irlanda	Control	T0	4.6
		T1	/
Asia	Control	T0	0.7
		T1	0.6
Maicol	Control	T0	0.6
		T1	0.6

## Data Availability

The data presented in this study are available on request from the corresponding author.

## Appendix A

**Table A1:** Allopregnanolone concentrations detected through hair sample analysis in ascending order of concentration.

Name	Group	Coat	Sex	Sampling Date	Sampling Type	Diff. Date T0–T1	Collection Site	Matrix	Allopregnanolone (pg/mg)
Maicol	Control	white and brown	MN	13/07/23	T0	0	left foreleg	hair	0.6
Asia	Control	white and chestnut	FS	20/08/23	T1	38	left foreleg	hair	0.6
Maicol	Control	white and brown	MN	20/08/23	T1	38	left foreleg	hair	0.6
Asia	Control	white and chestnut	FS	13/07/23	T0	0	left foreleg	hair	0.7
Cento	Case	white	MN	31/03/22	T0	0	left foreleg	hair	0.8
Cento	Case	white	MN	28/04/22	T1	28	left foreleg	hair	1.2
Ivry	Case	brown	FS	31/03/22	T0	0	left foreleg	hair	1.4
Ian	Case	cream	MN	11/03/23	T0	0	left foreleg	hair	1.9
Ivry	Case	brown	FS	28/04/22	T1	28	left foreleg	hair	2.0
Glitter	Case	black	MN	31/03/22	T0	0	right foreleg	hair	2.4
Parma	Control	cream	FS	31/03/22	T0	0	left foreleg	hair	2.7
Rocky	Control	black and tan	MN	31/03/22	T0	0	left foreleg	hair	3.0
Irina	Case	fawn	FS	07/05/23	T1	57	left foreleg	hair	3.1
Glitter	Case	black	MN	28/04/22	T1	28	right foreleg	hair	3.3
Parma	Control	cream	FS	28/04/22	T1	28	left foreleg	hair	3.4
Irina	Case	fawn	FS	11/03/23	T0	0	left foreleg	hair	3.4
Rocky	Control	black and tan	MN	28/04/22	T1	28	left foreleg	hair	4.0
Ian	Case	cream	MN	07/05/23	T1	57	left foreleg	hair	4.2
Irlanda	Control	fawn	FS	27/05/22	T0	0	right foreleg	hair	4.6
Irlanda	Control	fawn	FS	28/06/22	T1	32	right foreleg	hair	/
